# SIKVAV-Modified Chitosan Hydrogel as a Skin Substitutes for Wound Closure in Mice

**DOI:** 10.3390/molecules23102611

**Published:** 2018-10-11

**Authors:** Xionglin Chen, Xiaoming Cao, He Jiang, Xiangxin Che, Xiaoyuan Xu, Baicheng Ma, Jie Zhang, Tao Huang

**Affiliations:** 1 Department of Histology & Embryology and Medical Genetics, School of Basic Medical Sciences, Jiujiang University, Jiujiang 332000, China; 13879258264@163.com (H.J.); xuxy33@tom.com (X.X.); bcmnku@163.com (B.M.); bailihongchen@163.com (J.Z.); huang99037@163.com (T.H.); 2 Department of Anatomy, School of Basic Medical Sciences, Jiujiang University, Jiujiang 332000, China; cxm91016@163.com

**Keywords:** SIKVAV, chitosan hydrogel, skin wound healing, angiogenesis, growth factor

## Abstract

Skin wound healing is a complex and dynamic process that involves angiogenesis and growth factor secretion. Newly formed vessels can provide nutrition and oxygen for skin wound healing. Growth factors in skin wounds are important for keratinocytes and fibroblasts proliferation, epithelialization, extracellular matrix remodeling, and angiogenesis, which accelerate skin wound healing. Therefore, treatment strategies that enhance angiogenesis and growth factors secretion in skin wounds can accelerate skin wound healing. This study investigated the effects of a SIKVAV (Ser-Ile-Lys-Val-Ala-Val) peptide-modified chitosan hydrogel on skin wound healing. Hematoxylin and eosin (H&E) staining demonstrated that the SIKVAV-modified chitosan hydrogel accelerated the re-epithelialization of wounds compared with that seen in the negative and positive controls. Masson’s trichrome staining showed that more collagen fibers were deposited in the skin wounds treated with the SIKVAV-modified chitosan hydrogel than in the negative and positive controls. Immunohistochemistry assays demonstrated that more myofibroblasts were deposited and more angiogenesis occurred in skin wounds treated with the SIKVAV-modified chitosan hydrogel than in the negative and positive controls. In addition, ELISA assays showed that the SIKVAV-modified chitosan hydrogels promoted the secretion of growth factors in skin wounds. Taken together, these results suggest that the SIKVAV-modified chitosan hydrogel has the potential to be developed as synthesized biomaterials for the treatment of skin wounds.

## 1. Introduction

The skin is the largest organ of the human body, acting as a protective barrier against external disturbances. Intact skin prevents pathogen intrusion, infection, water, and electrolyte loss, and regulates the body temperature [1,2]. Large-area full-thickness skin defects can cause serious clinical problems such as severe infections and shock caused by substantial water and electrolytes loss, which can potentially result in death. Skin wound healing is a complex and dynamic process that involves coagulation, inflammation, angiogenesis, epithelial regeneration, granulation tissue formation, extracellular matrix (ECM) deposition, and tissue remodeling [3]. Therefore, a comprehensive understanding of the mechanism of skin wound healing is required to determine the most effective treatment [4]. The current skin wound treatment strategies include cytokine therapy, stem cell therapy, autograft, allograft, xenotransplantation, and tissue engineered skin substitutes [5]. Biomaterial-based wound dressings have recently drawn the attention of researchers.

Skin wound healing involves many cells (such as keratinocytes, stem cells, inflammatory cells and fibroblasts), a variety of growth factors (such as EGF (epidermal growth factor), bFGF (basic fibroblast growth factor), TGF-β1 (transforming growth factor beta-1), VEGF (vascular endothelial growth factor), and PDGF (platelet-derived growth factor)) and the ECM [3]. These growth factors promote the synthesis of ECMs and fibers by fibroblasts, thereby accelerating skin wound healing. EGF, which is produced by platelets, macrophages and fibroblasts acts on keratinocytes to promote the re-epithelialization of skin wounds. Studies have shown that EGF accelerates skin re-epithelization by promoting the proliferation and migration of keratinocytes in the process of acute wound repair [6]. The growth factor bFGF is secreted by keratinocytes, fibroblasts, vascular endothelial cells, smooth muscle cells, and mast cells. Studies have shown that in acute trauma, bFGF plays a critical role in granulation tissue formation, re-epithelialization, and tissue remodeling and promotes the synthesis of the ECM and fibers by fibroblasts [7]. TGF-β1 is secreted by keratinocytes, fibroblasts, macrophages, and platelets and plays an important role in the process of wound healing by triggering inflammatory reactions, angiogenesis, re-epithelialization and connective tissue regeneration [8]. VEGF, which is produced by vascular endothelial cells, keratinocytes, fibroblasts, platelets, neutrophils, and macrophages, promotes the proliferation and migration of vascular endothelial cells, thereby accelerating the formation of new blood vessels [9].

ECM consists of fibronectin, elastin, proteoglycan, and laminin. Laminin can promote cell proliferation and migration, regulate cell maturation and differentiation, and participates in cell signaling conduction [10]. Laminin is a Y-shaped structure that is formed by the binding of one α-peptide chain and two β-peptides through a sulfhydryl bond [11]. The α-peptide chain contains the sequence Ser-Ile-Lys-Val-Ala-Val (SIKVAV). Studies have shown that the peptide SIKVAV can promote fibroblasts, vascular smooth muscle cells, and vascular endothelial cells, as well as tumor cell adhesion, proliferation, and migration [12,13]. These physiological properties of endothelial cells are crucial for the formation of blood vessels in vivo. The peptide SIKVAV promotes bone marrow mesenchymal stem cell adhesion and differentiation into adipocytes and osteoblasts [14]. A previous study showed that SIKVAV covalently bound to poly-l-lactic acid promoted the regeneration of neurites in cerebellar stem cells in rats [15]. Hashimoto et al. [13] synthesized a composite wound dressing composed of the peptide SIKVAV covalently bound to sodium alginate that was able to promote skin wound healing. Therefore, the peptide SIKVAV shows potential as an effective therapeutic modality in the process of skin wound healing.

Chitosan is a natural cationic polymer with a high concentration of hydroxyl and the primary amino groups [16], which have been widely used in drug delivery, pharmaceuticals, hemostasis, antibacterial activity, wound healing, tissue engineering scaffolding, biotechnology, agriculture, and environmental protection applications, due to its good adsorption, drug loading and antibacterial properties [17,18,19]. Chitosan has been shown to accelerate wound healing by enhancing the function of inflammatory cells, macrophages, and fibroblasts [20]. Lefler et al. [21] treated skin wounds with a bFGF-containing chitosan dressing, and the results showed that the composite dressing could promote epithelial regeneration and inhibit bacterial growth. Dai et al. [22] treated mice with infected wounds with chitosan acetate bandages, showing that the material could inhibit bacterial growth. Chitosan is safe, biocompatible, biodegradable, and antiallergenic with antimicrobialand wound healing properties, and it can be synergistically combined with the peptide SIKVAV, thereby enhancing its angiogenesis and wound healing effects.

In our study, we successfully synthesized a composite hydrogel dressing composed of chitosan and the peptide SIKVAV. The hydrogel showed highly significant skin wound contraction and rapid wound regeneration effects in skin excision wounds in mice. Based on the potential wound healing properties of chitosan and the peptide SIKVAV, the present study evaluated its wound healing activity and sought to understand the healing mechanism of SIKVAV-modified chitosan hydrogel in a mouse skin wound defect model.

## 2. Materials and Methods

### 2.1. Materials

Chitosan (85% deacetylated with a molecular weight of 100,000 G/mol) was purchased from Golden-Shell Pharmaceutical Co., Ltd. (Yuhuan, China). Methacrylic Anhydride was purchased from the APC Chemicals Company (Montreal, PQ, Canada). 3-(Maleimid) Opropionic Acid *N*-Hydroxysuccinimide Ester (SMP; 97%) was purchased from Polysciences Corporation (Tamil Nadu, India). *N*,*N*,*N*,*N*-Tetramethylethylenediamine (TEMED), Ammonium Persulfate (APS), and Dimethylformamide (DMF) were purchased from Sigma-Aldrich (Guangzhou, China). The SIKVAV Peptide was obtained from Peptide Biotech Co., Ltd. (Shanghai, China). Sodium Pentobarbital was purchased from Aladdin (Guangzhou, China). The ELISA Test Kits for VEGF, EGF, TGF-B1, and Bfgf were obtained from the Shanghai Lichen Biotechnology Company (Shanghai, China). The CD31 Monoclonal Antibody was purchased from Dako (Guangzhou, China). The Alpha-Smooth Muscle Actin (A-SMA) Polyclonal Antibody, Biotinylated Secondary Antibody, and Streptavidin-Biotin Complex (SABC) Detection Kits were obtained from Wuhan Boster Biological Engineering Co., Ltd. (Wuhan, China). 

### 2.2. Synthesis of the SIKVAV-Modified Chitosan Hydrogel

The SIKVAV-modified chitosan hydrogel was prepared as described in our previous reports [23,24]. Briefly, methacrylic anhydride was added to a chitosan solution containing 3% glacial acetic acid and the mixture was then dialyzed to obtain unsaturated chitosan. The 3-Maleimidopropionic acid-*N*-succinimide ester was dissolved in dimethylformamide (DMF) and added to the unsaturated chitosan solution, which was subsequently stirred at room temperature overnight, dialyzed, and freeze-dried to obtain SMP-modified unsaturated chitosan. The peptide SIKVAV was added to the SMP-modified unsaturated chitosan solution and the resulting mixture was stirred at room temperature under nitrogen for 24 h, dialyzed and then lyophilized to obtain peptide SIKVAV-modified chitosan. Using a pipette gun to distribute them evenly across the solution surface, ammonium persulfate and a TEMED solution were added sequentially to the peptide SIKVAV-modified chitosan solution, which was then shaken to form a hydrogel. As a control, an unsaturated chitosan hydrogel without peptide SIKVAV modification was obtained using the same synthesis method of that of the SIKVAV-modified chitosan.

### 2.3. In Vivo Studies of Skin Wound Healing in Mice Using the SIKVAV-Modified Chitosan Hydrogel

Animal experiments were performed at the Animal Experimental Center of Jiujiang University and were approved by the Jiujiang University Ethics Committee (the project identification code: JJUEC20171002, date of approval: 8 October 2017), which strictly conforms to the NIH action guidelines for laboratory animal management and safety. Seventy-two female C57BL/6 mice aged 8–12 weeks were selected. The experimental mice were intraperitoneally injected with 1% sodium pentobarbital at 0.01 mL/g body weight, and the unilateral hair on the back of each mouse was removed after anesthesia. Subsequently, a 0.6 cm wound was made on the dorsal skin of each mouse with a hole punch. After the trauma model was established, the mice were randomly divided into 4 groups. The different wound treatment groups were as follows: no treatment (control group), wound treatment with a peptide SIKVAV solution (peptide SIKVAV group), wound covering with the chitosan hydrogel (chitosan group), and wound covering with the peptide SIKVAV-modified chitosan hydrogel (peptide SIKVAV + chitosan group). The chitosan hydrogel and the peptide-modified chitosan hydrogel were used after being sterilized by ultraviolet irradiation for half an hour before application. Each group was fed alone and freely. On days 3, 5, and 7 after trauma, a digital camera was used to record the wound size, and the proportion of the remaining wound area was calculated using the Equation (1):

Remaining area ratio of the wound (%) = St/So × 100%(1)

where So is the original area of the wound and St is the area of the wound that remains at the given time point.

### 2.4. Histological Observations

On days 3, 5, and 7 after trauma, the mice were euthanized by the intraperitoneal injection of 1% sodium pentobarbital at 0.05 mL/g body weight. Each wound and 5 mm of normal skin tissue around each wound were washed with PBS, fixed with 4% paraformaldehyde, washed again with PBS, dehydrated gradually using 70% to 100% ethanol, and embedded in paraffin. A 5 μm paraffin section was cut for each sample after which the tissue was stained according to the HE staining procedure and then subjected to trichromatic staining according to the Masson trichrome staining procedure.

### 2.5. Immunohistochemistry Assays

The 5 μm paraffin sections were deparaffinized, rehydrated and neutralized with 0.1 M citrate buffer solution (pH 6.0). To inactivate the endogenous enzymes, 10% H_2_O_2_ was applied for 10 min. The tissue sections were blocked with 5% BSA for 2 h and then incubated with a monoclonal goat anti-mouse α-SMA antibody at 4 °C overnight. After incubation, the tissue sections were reacted with SABC for 20 min, colored with 3,30-diaminobenzidine (DAB), stained with hematoxylin, and dehydrated using a gradient ethanol series. The tissue sections were soaked in xylene and then sealed with resin. Five randomly selected fields from each tissue section (three sections from three mice in each group) were observed microscopically (400×). Image-Pro Plus (Media Cybernetics, Rockville, MD, USA) was used to analyze the average optical density values for the α-SMA expression. Five randomly selected fields of view were examined for each group at each time point and were used to assess the average optical density value per unit area. Immunohistochemistry assays were also performed using the above methods to detect CD31 in the tissue samples.

### 2.6. ELISA Assay

EGF, bFGF, TGF-β1, and VEGF were measured on days 3, 5, and 7 after the trauma using ELISA kits. Wound tissue was extracted, washed with PBS, weighed, and then placed into normal saline at 10 µL saline per mg of tissue. After homogenization, the tissue was centrifuged at 3000 rpm for 10 min. Each wound tissue supernatant was immediately analyzed for TGF-β1, EGF, bFGF, and VEGF according to the instructions of each kit.

### 2.7. Statistical Analysis

The SPSS 20.0 software (International Business Machines Corporation, Guangzhou, China) was used to analyze the data. All measurement data are expressed as the mean ± standard deviation. For similar time points, the two samples were compared using an independent samples *t*-test, and multiple samples were compared by the one-way analysis of variance. A *p*-value of less than 0.01 (** *p* < 0.01) was used to indicate a statistically significant difference between groups. All experiments were performed at least three times.

## 3. Results

### 3.1. The SIKVAV-Modified Chitosan Hydrogel Promoted Skin Wound Contraction

**Figure 1:**
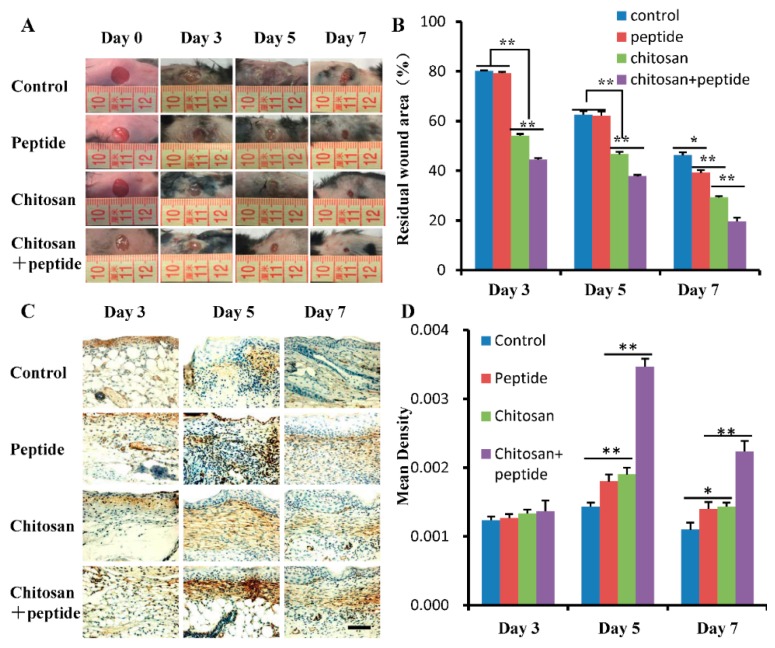
A SIKVAV-modified chitosan hydrogel promotes the contraction of skin wounds in mice: (**A**) Typical photoimages of the general wounds of control, SIKVAV, chitosan, and SIKVAV-modified chitosan group mice on days 3, 5, and 7 after trauma. (**B**) Statistical analysis of the residual wound percentage of mice in the control, SIKVAV, chitosan, and SIKVAV-modified chitosan groups (*n* = 6, * *p* < 0.05, and ** *p* < 0.01). (**C**) Immunohistochemistry showing the expression of α-SMA in the skin wounds of the control, SIKVAV, chitosan, and SIKVAV-modified chitosan group mice on days 3, 5 and 7 after trauma (scale bar: 50 μm). (**D**) Statistical analysis of α-SMA in the skin wounds of mice in the control, SIKVAV, chitosan, and SIKVAV-modified chitosan groups on days 3, 5 and 7 after trauma (*n* = 3, * *p* < 0.05, and ** *p* < 0.01).

A SIKVAV-modified chitosan hydrogel was successfully synthesized and applied as a wound dressing in a mouse model. The wound healing effect was quantified by determining the percentage of remaining wound area in each mouse wound. Overall, skin wound healing was better in the peptide SIKVAV-modified chitosan hydrogel group than in the control, peptide SIKVAV, and chitosan hydrogel groups (Figure 1A). As shown in Figure 1B, on day 3 after trauma, the remaining wounds area in the SIKVAV + chitosan group was smaller than those in the other three groups, but no significant difference was observed between the control group and the SIKVAV group. On days 5 and 7 after trauma, the remaining wound area in the SIKVAV + chitosan group was significantly smaller than those in the other groups, but no significant difference was observed in the wound area between the SIKVAV and control groups. These results show that the peptide SIKVAV-modified chitosan hydrogel can promote skin wound healing.

Myofibroblast traction promotes wound contraction in skin wounds. During the process of wound healing, the formation of granulation tissue is stimulated by traumatic factors and fibroblasts are transformed into contractile myofibroblasts to promote wound contraction. Further, α-SMA is highly expressed in the myofibroblasts. Therefore, we detected the expression of α-SMA in the skin wound tissues on days 3, 5, and 7 after trauma. The results are shown in Figure 1C. On day 3 after trauma, the α-SMA expression was weak in all wounds. The density of α-SMA in the tissue of each wound gradually increased until day 5 after trauma after which it gradually decreased to day 7 after trauma. A quantitative analysis of the α-SMA optical density values for the skin wound tissue at each time point are shown in Figure 1D; no significant differences were found between the control, peptide SIKVAV, chitosan, and SIKVAV + chitosan groups on day 3 post-trauma. However, the optical density of α-SMA in the skin wounds of the SIKVAV + chitosan group was significantly higher than those of the other groups on days 5 and 7 after trauma; statistically significant differences were observed between the SIKVAV group and the chitosan group at any time point.

### 3.2. The SIKVAV-Modified Chitosan Hydrogel Accelerated Skin Wound Re-Epithelialization

**Figure 2:**
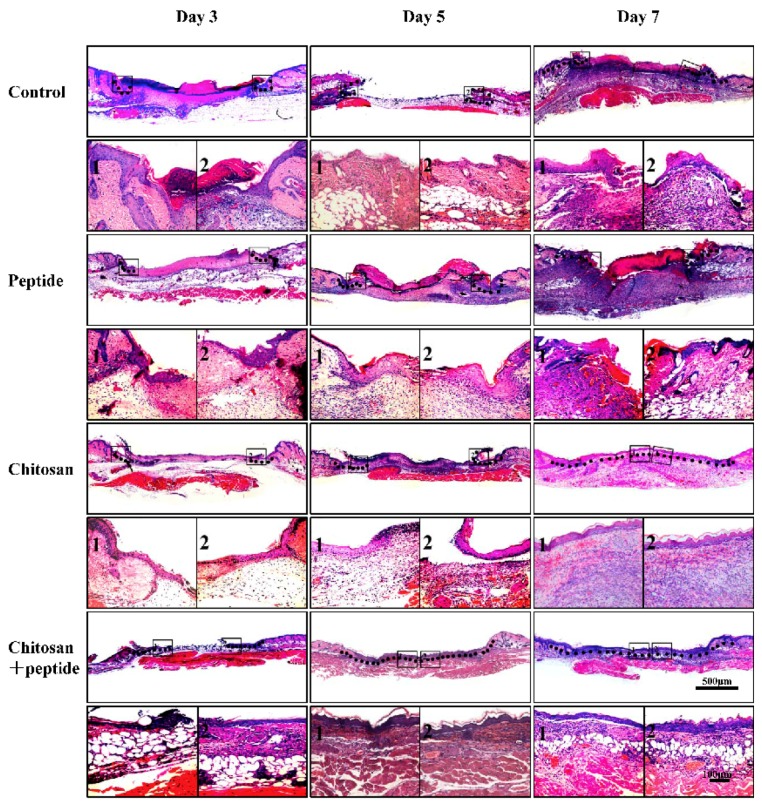
HE staining showed that the wounds were re-epithelialized on days 3, 5 or 7 after trauma in mice in the control, SIKVAV, chitosan, and SIKVAV-modified chitosan groups (scale bar: 50 μm). (The black frame indicates the junction between the re-epithelialization and non-re-epithelialization in the skin wound. The meaning of 1 and 2 means that the above corresponding picture is enlarged. 1 represents a magnified picture of the black frame on the left, and 2 represents a magnified picture of the black frame on the right.).

During wound healing, keratinocytes at the skin wound edge gradually cover the center of the wound via proliferation and migration, forming a new epithelium on the surface of proliferating granulation tissue. The SIKVAV-modified chitosan hydrogel promoted skin wound re-epithelialization (Figure 2). On day 3 after trauma, keratinocytes migrated a further distance from the wound edge in SIKVAV + chitosan group mice, than in the control, peptide, and chitosan mice groups. On day 5 after trauma, keratinocytes at the wound sites of mice in the SIKVAV + chitosan group completely covered the wound surface and then completed re-epithelialized. Keratinocytes did not completely cover the wound surfaces of mice in the control, peptide, and chitosan groups at this time. On day 7 after trauma, the chitosan group mice completed re-epithelization, but neither the control group nor the peptide group did. 

### 3.3. The SIKVAV-Modified Chitosan Hydrogel Promoted Skin Wound Angiogenesis 

**Figure 3:**
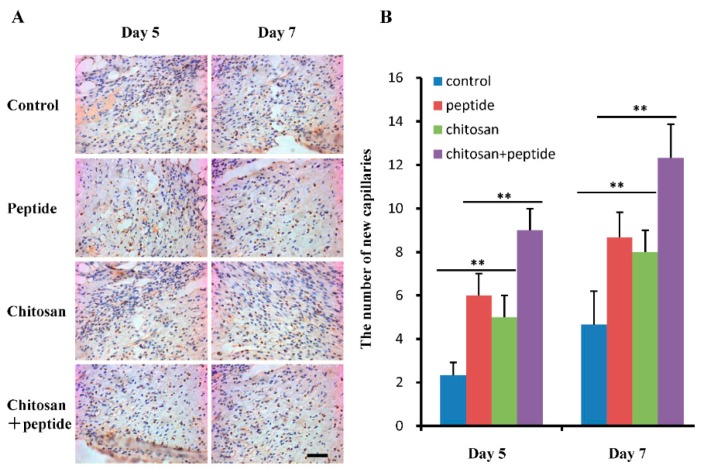
A SIKVAV-modified chitosan hydrogel promotes angiogenesis in skin wounds. (**A**) Immunohistochemical detection of CD31 expression in vascular endothelial cells of skin wounds on days 5 and 7 after surgery in control, SIKVAV, chitosan, and SIKVAV-modified chitosan group mice (scale bar: 50 μm). (**B**) Statistical analysis of new blood capillaries in control, SIKVAV, chitosan, and SIKVAV-modified chitosan group mice (*n* = 3, ** *p* < 0.01.).

New blood vessels in wounds provide nutrition for wound granulation tissue and new keratinocytes, which play an important role in wound healing. In this study, the expression of CD31 on the surface of endothelial cells in newly formed capillaries was analyzed by immunohistochemistry; the results are shown in Figure 3A. The proliferation of wounds in mice in the SIKVAV + chitosan group was better than that of the control, peptide, and chitosan mice groups. As shown in Figure 3B, the number of newly formed capillaries in the SIKVAV + chitosan group mice was significantly higher than that in mice in the chitosan, peptide, and control groups on day 5 after trauma. However, no significant difference was observed between the SIKVAV and chitosan groups. On day 7 after trauma, significantly more newly formed capillaries were apparent in the SIKVAV + chitosan group mice than in the mice in the other groups, while no statistically significant difference was found between the chitosan, and SIKVAV groups. These results demonstrate that the peptide SIKVAV-modified chitosan hydrogel can promote angiogenesis in skin wounds.

### 3.4. The SIKVAV-Modified Chitosan Hydrogel Promoted Skin Wound Collagen Synthesis 

**Figure 4:**
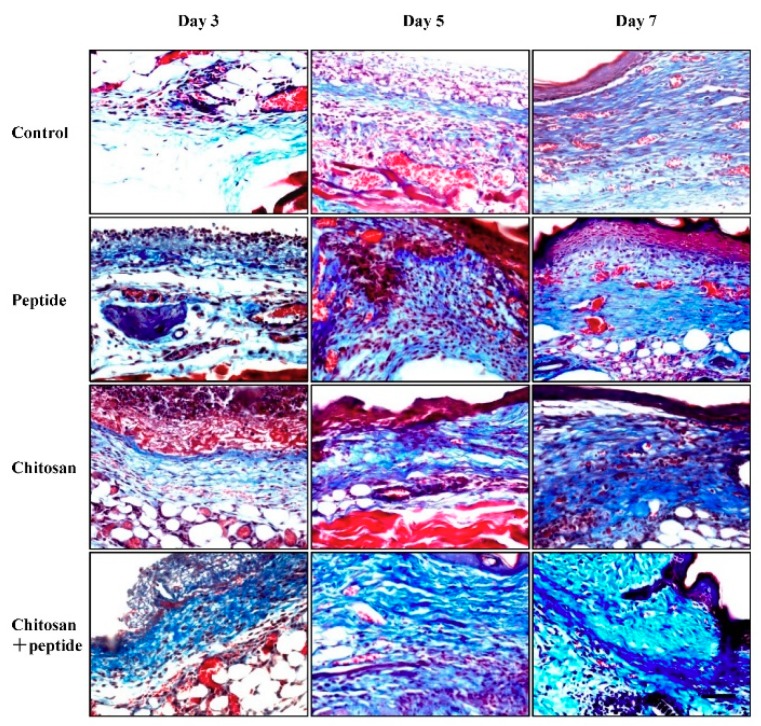
Masson trichrome staining showing the proliferation of new collagen fibers on days 3, 5 or 7 post-trauma in mice in the control, SIKVAV, chitosan, and SIKVAV-modified chitosan groups (scale bar: 50 μm).

Collagen synthesis plays a critical role in the process of skin wound healing, as it provides scaffolds for wound-healing cells and regenerative blood vessels, thus promoting wound healing. In this study, Masson trichrome staining was used to observe collagen in skin wounds. As shown in Figure 4, on day 3 after trauma, more nascent collagen fibers were observed in the skin wound granulation tissue of mice in the SIKVAV + chitosan group, while fewer collagen fibers were observed in the control, peptide, and chitosan group mice. On day 5 after trauma, the number of new collagen fibers increased in the skin wounds in the SIKVAV + chitosan group mice, while fewer collagen fibers were observed in the skin wounds of mice in the control, SIKVAV peptide, and chitosan groups. On day 7 after trauma, more nascent collagen fibers were found in the skin wounds of mice in the SIKVAV + chitosan group. At this time point, the number of collagen fibers began to increase in the skin wounds of mice in the SIKVAV and chitosan groups, but fewer fibers were found in the control group mice. These results indicate that the peptide SIKVAV-modified chitosan hydrogel can promote the deposition of wound collagen fibers to accelerate skin wound healing.

### 3.5. The SIKVAV-Modified Chitosan Hydrogel Promoted the Secretion of Growth Factors in Skin Wounds

**Figure 5:**
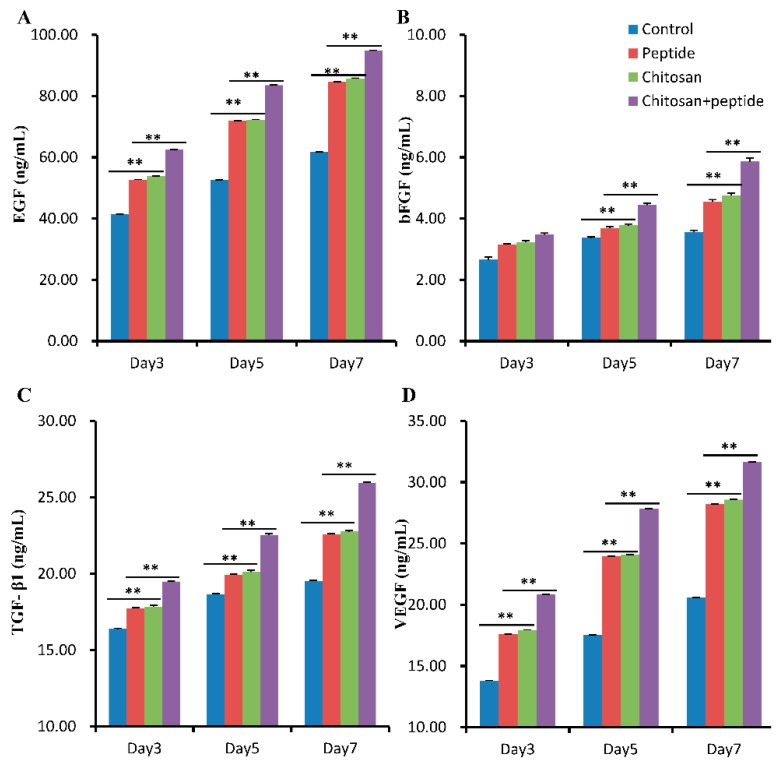
An ELISA assay detected the secretion of EGF (**A**), bFGF (**B**), TGF-β1 (**C**), and VEGF (**D**) in the skin wounds of mice on days 3, 5 and 7 after trauma in the control, SIKVAV, chitosan, and SIKVAV-modified chitosan groups (*n* = 3, ** *p* < 0.01.).

Skin wound healing involves a variety of growth factors that promote fibroblast secretion and synthesis, keratinocyte proliferation and migration, and endothelial cells proliferation and migration to form capillaries. ELISA assays were used to detect the secretion of growth factors in the skin wounds. As shown in Figure 5, the concentration of EGF, bFGF, TGF-β1, and VEGF had increasing trends on days 3, 5, and 7 after trauma. At each time point, the concentration of EGF, bFGF, TGF-β1, and VEGF in the skin wounds of mice in the SIKVAV-modified chitosan group were significantly higher than those of the control, peptide, and chitosan group mice. Furthermore, at each time point, the concentration of EGF, bFGF, TGF-β1, and VEGF in the skin wounds of mice in the chitosan and peptide groups were significantly higher than those in the control group mice; no significant difference was observed between the chitosan, and SIKVAV groups.

## 4. Discussion

Skin wound healing is a very complex process that includes coagulation, inflammation, cell proliferation, tissue formation (angiogenesis and connective tissue formation), and tissue remodeling [3]. After trauma, fibroblasts are activated and converted into myofibroblasts [25]. Myofibroblasts express α-SMA and promote skin wound contraction [20,26], which is mainly determined by the pulling effects produced by myofibroblasts. Fibroblasts synthesize extracellular collagen and ECM, which provide a scaffold for new blood vessels and the re-epithelialization of the wound during skin wound healing [20,26]. The results of this study showed that a SIKVAV-modified chitosan hydrogel promoted skin wound healing (Figure 1) and the deposition of new collagen fibers to a greater extent than those of the positive and negative control groups (Figure 4).

Angiogenesis plays an important role in cell proliferation, migration, differentiation, tissue formation and remodeling [27]. Alternatively, neovascularization can provide nutrition and oxygen for wound healing. Angiogenesis is regulated by a variety of growth factors, such as VEGF, bFGF, PDGF, and TGF-β1 [28]. Studies have shown that the peptide SIKVAV promotes endothelial cell adhesion, migration, and invasion [12,13] and physiological properties that are crucial for the formation of blood vessels in vivo. Studies have also demonstrated that the peptide SIKVAV can promote cell proliferation, and neurite outgrowth, as well as tumor cell metastasis and angiogenesis [12,13,29]. Therefore, the peptide SIKVAV shows potential as an effective treatment modality for skin wound healing. CD31 is a 130 kDa transmembrane glycoprotein that is found on the surface of platelets, monocytes, neutrophils, and some types of T-cells, as well as on the endothelial cells of new blood vessels [30]. Furthermore, CD31 is involved in angiogenesis and is primarily used to demonstrate the presence of endothelial cells in histological tissue sections, which can help to evaluate the degree of angiogenesis in healing tissue. The results of our study demonstrated that a SIKVAV-modified chitosan hydrogel promoted the secretion of growth factors (Figure 5) and angiogenesis compared to those in the positive and negative control groups (Figure 3).

The growth factor EGF is produced by macrophages, fibroblasts, and platelets [31,32]. EGF is a keratinocyte mitogen that accelerates re-epithelization by stimulating keratinocytes around skin wounds to proliferate and migrate to the wound center [6]. Our studies showed that in vivo, the SIKVAV-modified chitosan hydrogel accelerated the secretion of EGF (Figure 5A). Furthermore, according to HE staining, re-epithelialization in the skin wounds was greater than that in the other groups (Figure 2). This significantly greater re-epithelialization of skin wounds due to SIKVAV-modified chitosan hydrogel treatment can be attributed to increased EGF secretion in the skin wounds.

Although biomaterials have made a great impact in skin wound healing, recent studies have shown that a variety of stem cells also play an important role in skin wound healing. Studies have shown that a variety of stem cells are involved in skin wound healing, including bone marrow mesenchymal stem cells, adipose stem cells, induced pluripotent stem cells, and so on [33]. These cells can promote skin wound healing through paracrine growth factors (such as TGF-β, bFGF, VEGF, etc.), and can be differentiated into effective cells such as keratinocytes, fibroblasts, and endothelial cells, which promote the skin wound healing through enhancing vascularization, granulation tissue formation and re-epithelialization [33].

An ideal wound dressing should possess the following properties: acceleration of healing by modulating cytokines and growth factors, promotion of cell proliferation and matrix deposition, improvement of re-epithelialization and reduction of water and electrolyte loss. Many cells, cytokines, and growth factors are involved in the different phases of skin wound healing. The peptide SIKVAV directly or indirectly stimulates the secretion of many cytokines and growth factors in skin wounds [23] that are important for the proliferation of keratinocytes and fibroblasts, re-epithelialization, extracellular matrix remodeling, and angiogenesis, resulting in accelerated skin wound healing. The peptide SIKVAV-modified chitosan hydrogel promoted better skin wound healing than that seen in the other three groups, as shown in Figure 1. The illustration in Figure 5 explains the possible healing mechanism of the peptide SIKVAV-modified chitosan hydrogel.

## 5. Conclusions

This study on skin wounds in mice indicated that a SIKVAV-modified chitosan hydrogel accelerated skin wound healing and re-epithelialization, as well as collagen deposition and angiogenesis. The SIKVAV-modified chitosan hydrogel promoted the secretion of growth factors in skin wounds in vivo. Therefore, these results demonstrate that SIKVAV-modified chitosan hydrogels are promising synthesized biomaterials for the treatment of skin wounds.
